# The Mizan meta-memory and meta-concentration scale for students (MMSS): a test of its psychometric validity in a sample of university students

**DOI:** 10.1186/s40359-018-0275-7

**Published:** 2018-12-18

**Authors:** Md. Dilshad Manzar, Abdulrhman Albougami, Mohammed Salahuddin, Peter Sony, David Warren Spence, Seithikurippu R. Pandi-Perumal

**Affiliations:** 1 Department of Nursing, College of Applied Medical Sciences, Majmaah University, Al Majmaah, 11952 Saudi Arabia; 2 Department of Pharmacy, College of Medicine and Health Sciences, Mizan-Tepi University (Mizan Campus), Mizan-Aman, Ethiopia; 3 Department of Biomedical Sciences, College of Medicine and Health Sciences, Mizan-Tepi University (Mizan Campus), Mizan-Aman, Ethiopia; 4 Independent researcher, 652 Dufferin Street, Toronto, ON M6K 2B4 Canada; 5 Somnogen Canada Inc, College Street, Toronto, ON Canada

**Keywords:** Affective disorders, Cognitive function, Consistency, Divergent validity, Factor analysis, Khat, Meta-concentration, Meta-memory, Validity

## Abstract

**Background:**

Predisposing factors for metacognitive dysfunctions are common in university students. However, there is currently no valid questionnaire instrument designed to assess metacognitive aspects including meta-memory and meta-concentration in students. To address this need, the present study investigated the psychometric validity of a brief questionnaire, the Mizan meta-memory and meta-concentration scale for students (MMSS) in university students.

**Materials and methods:**

A cross-sectional study with simple random sampling was conducted among students (*n* = 383, age = 18–35, body mass index = 21.2 ± 3.4 kg/m^2^) of Mizan-Tepi University, Ethiopia. MMSS, a socio-demographics questionnaire, and the Epworth sleepiness scale (ESS) were employed.

**Results:**

No ceiling/floor effect was seen for the MMSS global and its sub-scale scores. Confirmatory factor analysis showed that a 2-Factor model had excellent fit. Both, the comparative Fit Index (CFI) and goodness of fit index were above 0.95, while both the standardized root mean square residual and root mean square error of approximation (RMSEA) were less than 0.05, while χ^2^/df was less than 3 and PClose was 0.31. The 2-Factor MMSS model had adequate configural, metric, scalar, and strict invariances across gender groups as determined by a CFI > .95, RMSEA<.05, χ^2^/df < 3, non-significant *Δ*χ^2^ and/or ΔCFI≤.01. Good internal consistency (Cronbach’s alpha = 0.84, 0.80 and McDonald’s Omega =0.84, 0.82) was found for both subscales of the MMSS. No correlations between the MMSS scores and ESS score favored its divergent validity.

**Conclusion:**

The MMSS was found to have favorable psychometric validity for assessing meta-memory and meta-concentration among university students.

**Electronic supplementary material:**

The online version of this article (10.1186/s40359-018-0275-7) contains supplementary material, which is available to authorized users.

## Background

The mental process of metacognition is a growing subject of neuro-psychological research, with particular relevance for the processes of teaching and learning, and thus for the education system [1]. Metacognition is defined as awareness and cognition about one’s own cognitive processes [2]. Individuals’ perceptions of their internal mental states, as well as their self and non-self attributions, are determined by a set of affective and cognitive skills, broadly described as meta-cognitive abilities [3]. Metacognitive problems are associated with impairments to the affected person’s social functioning, which in turn decrease their quality of life as well as their ability to respond to treatment [3]. Metacognitive impairments are associated with affective disorders such as depression, stress, and anxiety [3–5]. However, all of these affective states are commonly reported to occur among university students across the world [6, 7]. Furthermore, substance use, such as alcohol consumption is generally associated with metacognitive dysfunctions, and is a prevalent activity among university students in many parts of the world [8]. It was recently found that the prevalence of alcohol consumption and chewing of khat, an indigenous psychoactive substance, was, respectively, 32.3 and 27.9% among Ethiopian university students [8]. These relationships among metacognitive dysfunctions, affective disorders, and substance use that are prevalent in student populations highlight the need for a tool to screen for dysfunctions in metacognition and its aspects among university students.

Meta-memory and meta-concentration are two very important dimensions of metacognition [9, 10]. Meta-memory and meta-concentration are associated with success in everyday functioning. Furthermore, there is an interaction effect between these two metacognitive aspects that is essential for success in daily routine activities [10]. Those with meta-cognitive and meta-memory deficits develop a protectionist approach to avoid challenging situations, thus affecting their capacity to deal with similar situations in the future, and thus having broadly detrimental effects for dealing with life problems and adjustment [9, 11]. There is a reciprocal relationship between meta-memory and other metacognitive characteristics such as vocabulary development and comprehension [12]. Meta-cognitive instructions have intermediate and delayed effects, which can manifest in improved mathematical achievement and improved cognitive regulation among students [13]. Various studies have suggested that knowledge about meta-memory can be acquired and may directly benefit the learning process in students [14]. Metacognitive abilities related to concentration i.e., meta-concentration, is one of the most important non-intellective psychological factor which can influence students’ performance, as indicated by grade point average [15]. At the present time, there is no questionnaire designed to measure these metacognitive aspects, either separately or in terms of their interactive effects, in student populations. It was thus felt that a brief, easily administered, and valid questionnaire would be of use to campus counselors, psychologists, and others. It was also felt that such a tool could help in the routine screening of the students.

We therefore investigated the literature on this subject for useful examples of instruments that could be adapted for use with students. A number of excellent psychometric instruments currently exist for diagnosing meta-memory and meta-concentration. These include commonly used questionnaires for metacognition such as the Metamemory in Adulthood (MIA) scale [16], which has 108 items, the Metacognition Questionnaire (MCQ), which has 65 items [17], and the Metacognition Questionnaire-30 (MCQ-30), which has 30 items [17]. These instruments, however, are primarily designed for use in medical or psychiatric settings, and while they tend to be exhaustively comprehensive, they can be cumbersome and time-consuming to administer. An exception to this generalization is a brief metacognition questionnaire,which was recently developed for use at the Charité - University Medicine Berlin [10]. The present investigators reviewed this questionnaire and used it as a guide for developing the questionnaire that is reported on here, although it has been modified to make it more appropriate for students. In this study, we present the psychometric properties of this adapted version of a brief meta-memory and meta-concentration questionnaire, which has been designed to suit the daily activities of university student populations.

## Methods

**Fig. 1:**
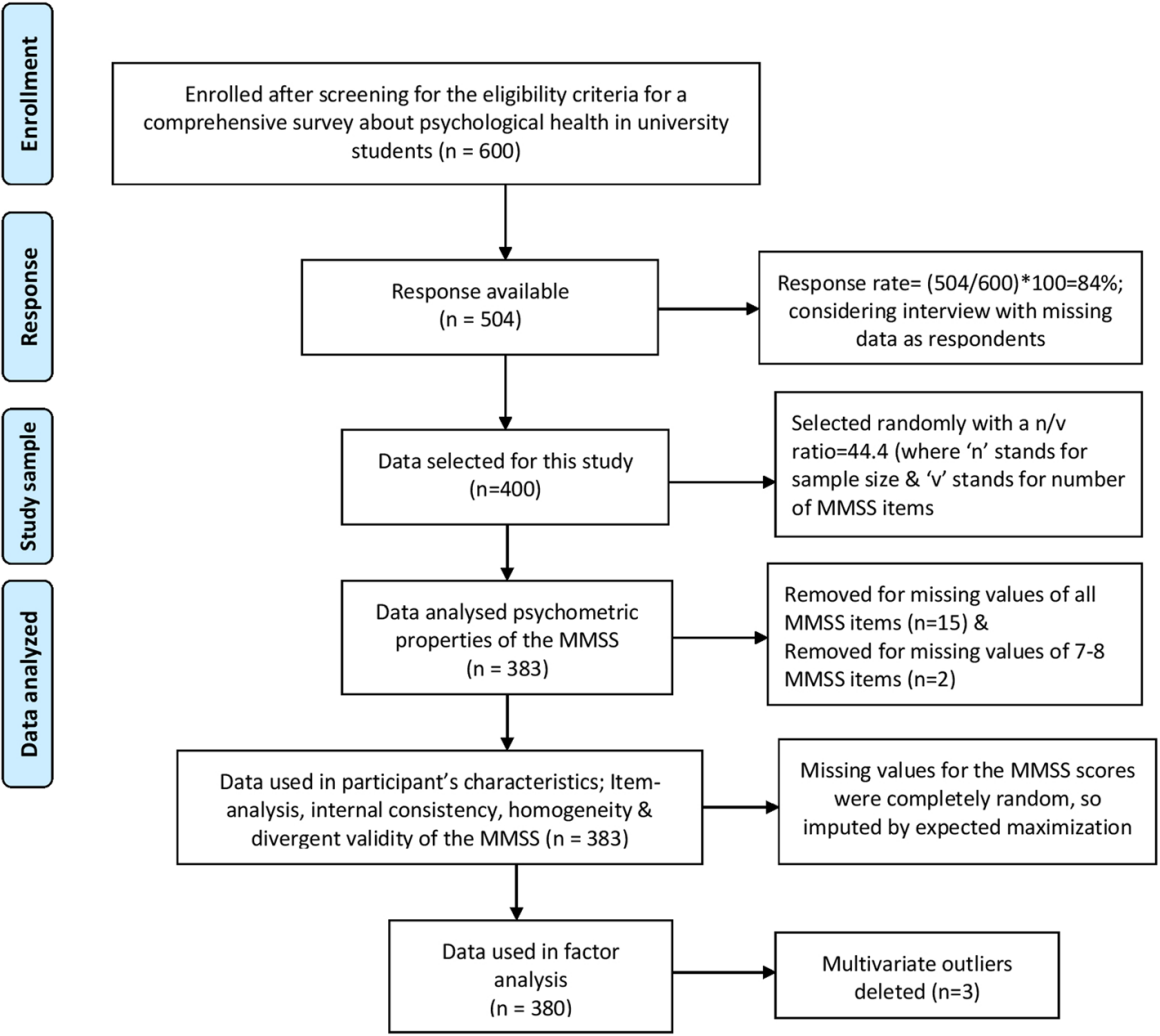
Schematic of study sample

The study presents findings of data taken (Fig. 1) from a cross-sectional study using simple random sampling method regarding psychological health and associated factors among university students carried out at the Mizan campus of the Mizan-Tepi University (MTU), Mizan-Aman, Bench Maji Zone, South Nation Nationalities Peoples Region, Ethiopia.

### Participants

Three hundred and eighty-three university students with an age range of 18–35 years and a body mass index of 21.2 ± 3.4 kg/m^2^ completed this study. Students with self-reported mental illness difficulties, such as a previous diagnosis of depression or psychosis that might have compromised the data quality were excluded. Similarly, those under the age of 18 years were not included because in such cases consent would have to have been obtained from their parents as well, a difficult requirement to fulfill inasmuch as many students were from remote regions of the country.

### Procedures

The Institutional Ethics Committee, College of Medicine and Health Sciences, Mizan-Tepi University approved the research. Guidelines for Good Clinical Practice and the norms of the 2002 Declaration of Helsinki (DoH) were followed. Informed written consent was provided by the participants after the objective and procedures of the study were explained to them. The Mizan meta-memory and meta-concentration scale for students (MMSS), a semi-structured socio-demographics questionnaire, plus the Epworth sleepiness scale (ESS) were employed. The questionnaire packages were administered in English because participating students belonged to different linguistic groups and had differing levels of proficiency for reading Amharic. Moreover, the study participants were students of a university in Ethiopia, where the medium of instruction is English. The instruments were administered to the participants at the university premises by those members of the team of investigators who were also part of the MTU faculty.

### The Mizan meta-memory and meta-concentration scale for students (MMSS)

#### Background and questionnaire conceptualization

As a first step for developing the scale, a panel of experts was brought together to discuss the objective of constructing a new tool for assessing meta-memory and meta-concentration in the target audience of university students. Panel members, who were drawn from the fields of psychometrics, physiology, medicine, statistics, and languages, were asked to develop scale items according to several criteria. Among these criteria priority consideration was given to the scale’s potential usability in survey research, response rate maximization, conciseness, and appropriateness as a preliminary screening tool. Following a detailed search of the literature which sought to gather previous experience regarding scale readability as well as comprehensibility, twelve items were generated. Some of the items were adapted from a metacognition assessment instrument developed by Klusmann and colleagues at the Department of Psychiatry, Charité - University Medicine Berlin [10]. The items measuring meta-memory were based on the questionnaire of meta-memory in adulthood developed by Dixon and colleagues [16]. The items measuring meta-concentration were based on the EURO-D, which was developed by Prince and colleagues [18]. The items of the original instrument were adapted to suit the metacognitive functions associated with the daily activities of students. None of items were reverse scored in our preliminary questionnaire.

#### Format and content validity

The panel of experts assessed and revised these items for relevance, comprehension and clarity. It was agreed to delete one of the items, ‘I am good at reasoning, planning activities, or solving problems’, after discussions because experts did not find it relevant to meta-memory and meta-concentration.

#### Field testing

An 11-item scale was finally developed and employed in an initial field test. This testing led to a decision to delete two items due to their significantly adverse effect on the overall internal consistency as determined by the Cronbach’s alpha test. These items were, ‘I have no issues of memory losses’ and ‘I have no difficulties related to concentration’.

#### Final tool: MMSS

The preliminary testing of the MMSS produced a brief questionnaire with nine items that assess two aspects of metacognition, i.e., meta-memory (five items) and meta-concentration (four items). The MMSS used in this study is shown in Additional file 1: Appendix I. The items are scored in the range of 1–5, where, ‘1’ stands for ‘strongly disagree’ and ‘5’ denotes ‘strongly agree’. Individual scores of the 9-items of the MMSS are linearly added to get the global score of the MMSS in the range of 9 and 45, where higher scores imply good meta-cognitive ability in areas of meta-memory and meta-concentration. Individual scores of the five items of the meta-memory subscale are linearly added to obtain the total score for this dimension. Similarly, scores for the four items of the meta-concentration subscale are added to get total score for this dimension.

### Epworth sleepiness scale

The ESS is an eight item questionnaire which is used to assess daytime sleepiness [19]. These 8-items have a four-point scale, where, ‘0’ indicates ‘would never nod off’, while, ‘3’ indicates a high chance of nodding off in eight different situations encountered in daily lives [19]. The scores of individual item scores are added to get the ESS total score in the range of 0 to 24. Increasing levels of daytime sleepiness are indicated by higher ESS scores [19].

### Socio-demographics questionnaire

A semi-structured socio-demographics questionnaire with nine items, one open ended and eight close ended, were used. Information concerning the respondent’s age, gender, ethnicity, alcohol use, khat use, smoking, use of tea/coffee, use of other beverages such as soft drinks and other fermented/non-fermented non-alcoholic indigenous drinks and presence of chronic conditions were collected. Height and weight were taken for assessing body mass index.

### Statistical analysis

Data analysis was performed by SPSS version 23.0, an add-on module called AMOS, and two plugins [20, 21]. Participants’ characteristics were examined using the mean (±SD), frequency, and percentage. Item analysis was performed by mean (±SD), skewness, kurtosis, percentage, Spearman’s item-Factor correlations, and the Cronbach’s alpha (if the item were deleted). The internal consistency of the responses was assessed by the application of the Cronbach’s alpha and the McDonald’s Omega test. Nunnally and Bernstein have suggested that during the initial stage of research, as in the case of questionnaire development, a Cronbach’s alpha of 0.70 is sufficient. However, the experimental research where emphasis is on quantitative aspect of correlation as well as the differences in mean, a Cronbach’s alpha of 0.80 may be desirable [22]. The internal homogeneity and divergent construct validity were evaluated by the Spearman’s correlation coefficient test.

Three multivariate outliers were identified, and hence deleted, for factor analysis following application of Mahalanobis distance testing (criterion of a = .001 with 9df, the critical χ^2^ = 33.72) (Fig. 1) [23]. Six of the MMSS items were skewed (Z score of Skewness≥ ± 3.29) (Table 1). All the items were retained without transformation inasmuch as a related instrument was found to be valid in German and Portuguese samples [10, 24].

**Table 1:** Descriptive statistics of the Mizan meta-memory and meta-concentration scale for students (MMSS) in university students

Items of the MMSS	Cronbach’s Alpha if Item Deleted		Item-Factor correlation		Mean ± SD	Skewness		Kurtosis		Percentage distribution across item scores					
	1-F	2-F	1-F	2-F		Statistic(SE)	z	Statistic(SE)	z	1	2	3	4	5	Missing value
BMMS-1	.81		.75^*^		3.44 ± 1.05	−.57(.12)	−4.53	−.31(.25)	−1.23	5.2	14.4	24.0	43.6	12.5	.3
BMMS-2	.81		.76^*^		3.53 ± 1.10	−.64(.12)	−5.13	−.33(.25)	−1.33	5.5	14.4	18.5	43.9	17.2	.5
BMMS-3	.83		.77^*^		3.38 ± 1.27	−.39(.12)	−3.13	−.93(.25)	−3.73	9.9	16.2	20.1	30.5	22.2	1.0
BMMS-4	.79		.80^*^		3.53 ± 1.09	−.68(.12)	−5.45	−.25(.25)	−.99	5.5	13.6	18.6	45.5	16.4	.5
BMMS-5	.80		.77^*^		3.44 ± 1.08	−.49(.12)	−3.89	−.39(.25)	−1.58	5.5	13.8	26.4	38.6	14.9	.8
BMCS-1		.72		.82^*^	3.35 ± 1.10	−.50(.12)	−3.98	−.47(.25)	−1.90	7.3	15.1	25.3	39.9	12.3	.0
BMCS-2		.74		.78^*^	3.25 ± 1.03	−.30(.12)	−2.43	−.48(.25)	−1.94	5.5	18.0	31.6	35.0	9.4	.5
BMCS-3		.79		.75^*^	3.38 ± 1.15	−.37(.12)	−2.96	−.58(.25)	−2.35	7.3	14.1	29.0	31.1	17.8	.8
BMCS-4		.74		.76^*^	3.41 ± 1.11	−.47(.12)	−3.77	−.32(.25)	−1.30	7.3	11.0	31.1	34.2	16.2	.3
1-F					17.32 ± 4.39	−.61(.12)	−4.90	−.06(.25)	−.24						
2-F					13.39 ± 3.47	−.38(.12)	−3.02	−.11(.25)	−.43						

*D* Standard deviation, *SE* Standard Error

*BMMS* Brief Meta-memory sub-scale, *BMCS* Brief Meta-concentration sub-scale, BMMS-1 to BMMS-5: items of BMMS, BMCS-1 to BMCS-4: items of BMCS

1-F: Meta-memory subscale; 2-F: Meta-concentration subscale

*p < .01

**Fig. 2:**
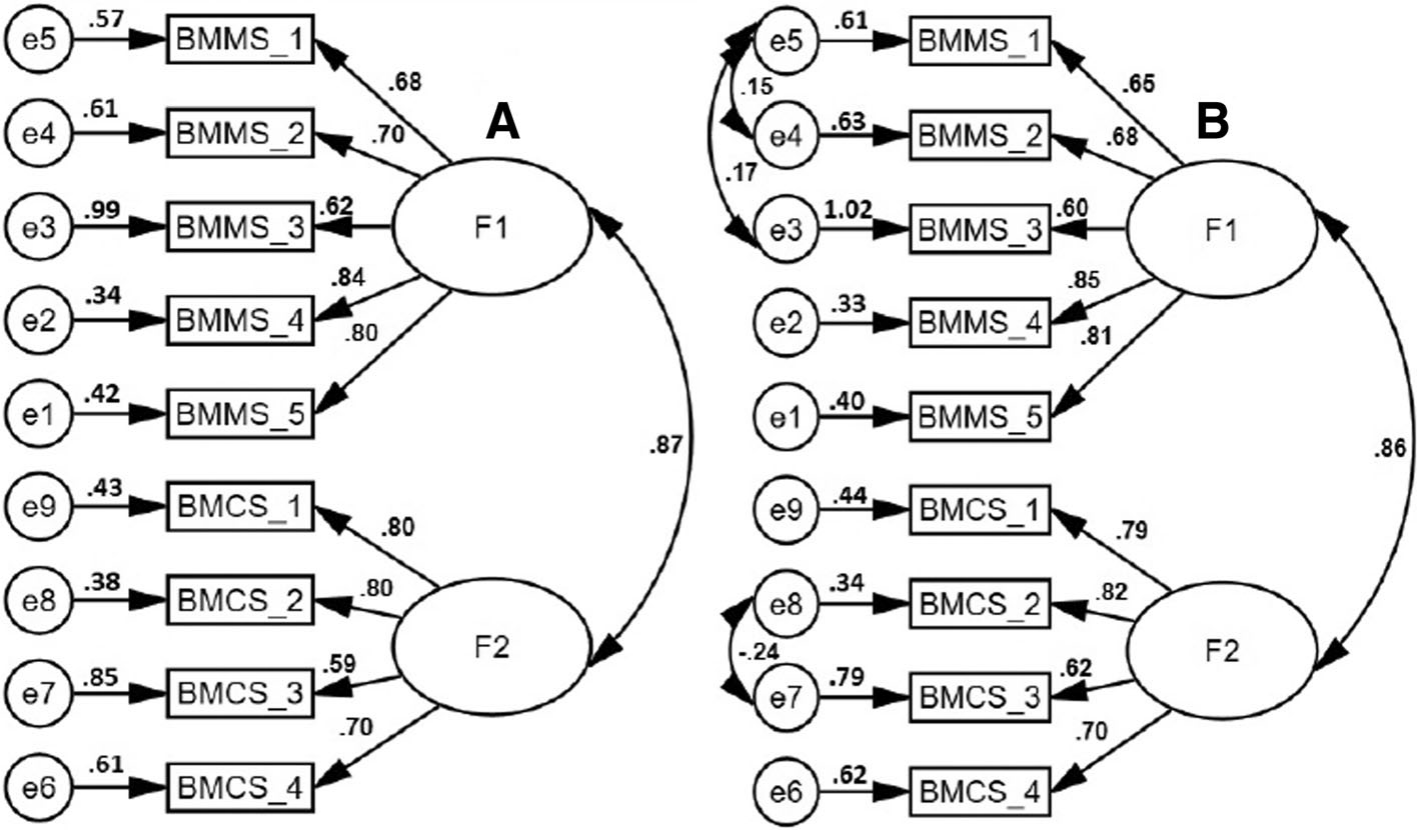
Confirmatory factor analysis models of the Mizan meta-memory and meta-concentration scale for students (MMSS) in university students. A: 2-Factor, B: 2-Factor model with incorporation of modification indices (correlated error terms). BMMS: Brief Meta-memory sub-scale; BMCS: Brief Meta-concentration sub-scale, bmms_1 to bmms_5: items of BMMS, bmcs_1 to bmcs_4: items of BMCS. All coefficients are standardized. *Ovals* latent variables, *rectangles* measured variables, *circles* error terms, *single-headed arrows* between *ovals* and *rectangles* factor loadings, *single-headed arrows* between *circles* and *rectangles* error terms. Amos does not display standardized values of uniqueness on the models; therefore models were manually edited to put numerical values taken from the Amos text output (Estimates→Scalars→Variances)

In view of the fact that six item scores were skewed a confirmatory factor analysis (CFA) using maximum likelihood extraction with bootstrapping was carried out. Modification indices (co-varying error terms) were employed to increase the fit during confirmatory factor analysis (CFA). The standardized loadings of the MMSS item scores on the respective factors were estimated. CFA was used to screen two 2-Factor models; model-A: a 2-Factor model based on theoretical considerations [10], and model-B: a 2-Factor model with incorporation of modification indices (co-varying error terms) (Table 2, Fig. 2). Multiple fit indices from different categories were employed according to recommended norms [23, 25, 26]. Analyses based on discrepancy functions, such as χ^2^, χ^**2**^/df and standardized root mean square residual (SRMR), absolute fit index, the goodness of fit index (GFI), tests comparing target model with the null model (such as the comparative fit index [CFI]), non-centrality indices (such as the root mean square error of approximation [RMSEA]), and PClose were employed [23, 27]. The findings for various tests, e.g., RMSEA (≤ .08), RMR (≤ 0.05) and χ^**2**^/df (≤3) indicated an acceptable fit [28]. For CFI and GFI a value greater than 0.95 implied an excellent fit [28]. A non-zero value of the PClose also indicated an acceptable fit [28]. Tests for evaluation of configural, metric/weak, scalar/strong and strict measurement invariance for the model validated by CFA were performed.

**Table 2:** Discriminant or divergent validity: Correlation of the Mizan meta-cognition scale for students (MMSS) scores with Epworth sleepiness scale (ESS) scores in university students

MMS scores	ESS score
BMMS-1	−.04
BMMS-2	−.11
BMMS-3	−.01
BMMS-4	−.05
BMMS-5	−.01
BMCS-1	−.11
BMCS-2	−.06
BMCS-3	−.06
BMCS-4	−.06
Meta-memory	−.07
Meta-concentration	−.11
Total score	−.04

*BMMS* Brief Meta-memory sub-scale, *BMCS* Brief Meta-concentration sub-scale, BMMS-1 to BMMS-5: items of BMMS, BMCS-1 to BMCS-4: items of BMCS

## Results

### Participants’ characteristics

Participants’ characteristics are shown in Table 3. The mean age was 21.2 ± 3.4 years, and students with normal BMI’s formed the largest subgroup, making up 66.1% of the sample (Table 3). Amhara and Oromo ethnicities together comprised the majority (59%) of the study population (Table 3). The self-reported prevalence of the use of alcohol, Khat and cigarettes were 10.2, 9.9 and 5.7%, respectively (Table 3). Nearly 1/10th, i.e., 11.5% of the sample, reported having chronic medical conditions, including AIDS, hepatitis-A, hepatitis-B, hypertension, diabetes mellitus I/II, and tuberculosis (Table 3). It was observed that a high mean MMSS global score of 30.71 ± 7.29 occurred in the study population (Table 3).

**Table 3:** Participant characteristics

Characteristics	Mean ± SD/frequency
Age (yr)	20.97 ± 1.83
BMI (Kg/m^2^)	
Underweight	51 (13.3)
Normal	253 (66.1)
Over-weight	19 (5.0)
Obese	9 (2.3)
Unreported	51 (13.3)
Gender	
Male	261 (68.1)
Female	103 (26.9)
Unreported	19 (5.0)
Ethnicity	
Amhara	142 (37.1)
Tigray	8 (2.1)
Oromo	84 (21.9)
Keffa	2 (0.5)
Bench	3 (0.8)
Others	49 (12.8)
Unreported	95 (24.8)
Substance use	
Alcohol	
Yes	39 (10.2)
No	339 (88.5)
Unreported	5 (1.3)
Khat	
Yes	38 (9.9)
No	337 (88.0)
Unreported	8 (2.1)
Smoking	
Yes	22 (5.7)
No	356 (93.0)
Unreported	5 (1.3)
Tea/Coffee	
Yes	343 (89.6)
No	40 (10.4)
Other beverages	
Yes	254 (66.1)
No	99 (25.8)
Unreported	30 (7.8)
ESS	6.9 ± 4.7
Presence of Chronic conditions	
No	215 (56.1)
Yes	44 (11.5)
Unreported	124 (32.4)
MMSS global score	30.71 ± 7.29

*SD* standard deviation, *ESS* Epworth sleepiness scale

Chronic health conditions like AIDS, Hepatitis-A, Hepatitis-B, Hypertension Diabetes Mellitus I/II, Tuberculosis, others

*MMSS* Mizan meta-memory and meta-concentration scale for students

### Preliminary item analysis

The descriptive analysis of the MMSS scores is shown in Table 1. There were 0–1% missing values for the MMSS item scores in the final study sample. Little’s test [χ^2^ = 65.98 (df = 62), *p* < 0.34] indicated that the missing values for MMSS scores were completely random. Missing values were dealt with by adding in the expected maximization because it is a method of choice irrespective of sample size, the proportion of data missing, and distribution characteristics [29]. None of the MMSS item scores showed a floor effect; the lowest score occurred in less than 15% of the sample [30, 31]. However, five items, i.e., BMMS-2, BMMS-3, BMMS-4, BMCS-3,and BMCS-4 demonstrated a ceiling effect, i.e., the highest scores were achieved by more than 15% of the respondents [30, 31]. The MMSS global score did not demonstrate any significant problems in terms of ceiling/floor effects, with 0.5% reporting the lowest score of 9 and 0.8% reporting the highest score of 45. The meta-memory score did not demonstrate any significant problems in terms of ceiling/floor effects, with 1.0% reporting the lowest score of 5 and 1.8% reporting the highest score of 25. The meta-concentration score did not demonstrate any significant problems in terms of ceiling/floor effects, with 0.8% reporting the lowest score of 4 and 4.7% reporting the highest score of 20.

### Factor analysis

#### Measures assessing adequacy, suitability and factorability of the MMSS scores

The diagonal elements of the anti-image correlation matrix of the MMSS item scores were either 0.89 or above, satisfying the condition for factor analysis (Table 4) [32]. The MMSS item scores had an excellent degree of shared variance, as indicated by a Kaiser-Meyer-Olkin Test of sampling adequacy of 0.91 (Table 4) [32]. The MMSS item scores had linear combinations necessary for factor analysis, as suggested by a significant Bartlett’s test of sphericity (Table 4) [32]. There was neither an issue of singularity nor of the multicollinearity as required for factor analysis in the MMSS item score, because the determinant of the correlation matrix was greater than 0.00001 and less than 1 (Table 4) [32]. A threshold for variance was derived from the common factors as determined by a range of 0.34 to 0.65 for the communality (Table 4), therefore all the MMSS items were retained for the factor analysis [33]. None of the inter-item correlations were less than 0.3 (*r* = 0.37–0.71, *p* < 0.01), therefore ideal conditions were found for the factorability of the MMSS item score correlation matrix [34] (Table 5).

**Table 4:** Sample size adequacy measures of the Mizan meta-cognition scale for students (MMSS) in university students

Measures	Values
Anti-image matrix	0.89–0.94
Bartlett’s test of Sphericity	*Χ*^2^ (df = 36), *p* < 0.001
Determinant	0.016
Kaiser-Meyer-Olkin Test of Sampling Adequacy (KMO)	0.91
Communality	0.34–0.65

**Table 5:** Inter-item Correlation matrix of the Mizan meta-memory and meta-concentration scale for students (MMSS) in university students

	BMMS-1	BMMS-2	BMMS-3	BMMS-4	BMMS-5	BMCS -1	BMCS -2	BMCS -3	BMCS -4
BMMS-1		.51^*^	.49^*^	.52^*^	.46^*^	.43^*^	.42^*^	.42^*^	.32^*^
BMMS-2			.44^*^	.56^*^	.53^*^	.46^*^	.45^*^	.41^*^	.42^*^
BMMS-3				.49^*^	.46^*^	.42^*^	.35^*^	.45^*^	.32^*^
BMMS-4					.68^*^	.55^*^	.56^*^	.39^*^	.47^*^
BMMS-5						.47^*^	.57^*^	.37^*^	.52^*^
BMCS-1							.62^*^	.50^*^	.49^*^
BMCS-2								.38^*^	.53^*^
BMCS-3									.44^*^
BMCS-4									

^*^p < 0.01

*BMMS* Brief Meta-memory sub-scale, *BMCS* Brief Meta-concentration sub-scale, BMMS-1 to BMMS-5: items of BMMS, BMCS-1 to BMCS-4: items of BMCS

#### Confirmatory factor analysis (CFA)

Table 6 shows the goodness of fit statistics of the models screened in the CFA of the MMSS scores in the university students. Both models had either an excellent or an acceptable fit, i.e., CFI and GFI > .95, SRMR and RMSEA<.08 and χ^2^/df < 3 and PClose> 0 [28].

**Table 6:** Fit statistics of the Mizan meta-memory and meta-concentration scale for students (MMSS) models in university students

Models	CFI	GFI	SRMR	RMSEA	χ^2^	df	*p*	χ^2^/df	PClose
A	.97	.95	.04	.07(.05–.09)	77.95	26	<.001	3.00	.02
B	.98	.97	.03	.06(.03–.08)	49.72	23	.001	2.16	.31

A: 2-Factor, B: 2-Factor model with incorporation of modification indices (correlated error terms)

*CFI* Comparative Fit Index, *GFI* Goodness of fit index, *SRMR* Standardized root mean square residual, *RMSEA* root mean square error of approximation

#### Measurement invariance of model-B among gender groups

The configural invariance of Model-B was excellent as indicated by values of the fit indices (χ^2^/df < 2, CFI > .95, RMSEA (CI) < .05, when groups were estimated without constraints (Table 7). Chi-square testing did not reveal significant differences ([Δχ^2^(df) = 10.988 (7), *p* = .139] and ΔCFI <.01) between the model constrained for loadings and the fully unconstrained model, thus supporting metric or weak invariance of the Model-B, across gender groups (Table 7) [35]. Strong or scalar invariance of model-B was indicated by a finding of non-significance following chi-square testing ([Δχ^2^(df) = 14.234 (9), *p* = .114] and ΔCFI <.01) between models constrained for loadings and models constrained for intercepts (Table 7) [35]. Models constrained for residuals and models constrained for intercepts showed significant chi-square differences ([Δχ^2^(df) = 53.024 (15), *p* < .001] but ΔCFI <.01) (Table 7) [35].

**Table 7:** Measurement invariance of the 2-Factor model among gender groups of the Mizan meta-memory and meta-concentration scale for students (MMSS) in university students

	*Χ* ^2^	df	*P* value	*Χ*^2^/df	CFI	RMSEA	*Χ*^2^ difference test statistics			ΔCFI
							*ΔΧ* ^2^	Δdf	*P* value	
2-Factor model: MMSS										
Equal form	82.868	46	.001	1.801	.977	.047				
Metric invariance-Equal loadings	93.856	53	.000	1.771	.974	.046	10.988	7	.139	−.001
Scalar invariance-Equal intercepts	108.091	62	.000	1.743	.971	.046	14.234	9	.114	.000
Strict invariance-Equal factor variances	161.115	77	.000	2.092	.947	.055	53.024	15	.000	+.009

#### Internal consistency and homogeneity

The Cronbach’s alpha for the meta-memory and meta-concentration subscales were 0.84 and 0.80, respectively (Table 8). The McDonald’s Omega for the meta-memory and meta-concentration subscales were 0.84 and 0.82, respectively (Table 8). Item-Factor score correlations for the meta-memory subscale ranged between *r* = 0.75 (*p* < .01) and *r* = 0.80 (*p* < .01) (Table 1). Item-Factor score correlations for meta-concentration subscale ranged between *r* = 0.75 (*p* < .01) and *r* = 0.82 (*p* < .01) (Table 1). Inter-item correlations ranged between *r* = 0.32 (*p* < .01) and *r* = 0.68 (*p* < .01) (Table 5).

**Table 8:** Internal consistency: Cronbach’s alpha and McDonald’s Omega of the 2-Factor model of the Mizan meta-memory and meta-concentration scale for students (MMSS) in Ethiopian university students

	Cronbach’s alpha	McDonald’s Omega
Meta-memory	0.84	0.84
Meta-concentration	0.80	0.82

#### Divergent construct validity

There was no significant correlation between the ESS score and MMSS scores.

## Discussion

This is the first study to carry out a psychometric validation of an instrument for measuring two important aspects of meta-cognition i.e., meta-memory and meta-concentration, in a student population. The study found sufficient psychometric validation of the MMSS to support the conclusion that this instrument measures what it is intended to measure. This was evidenced by the absence of findings of major issues in terms of ceiling/floor effect, favorable item discrimination, factorial validity and measurement invariance across gender groups, internal consistency, and divergent validity.

### Preliminary item analysis

There was some concern about the ceiling effect in five item scores of the MMSS; the presence of this phenomenon could possibly affect the responsiveness and discriminative validity of this instrument for the highest score of these items [30]. The MMSS items are scored in such a way that normal behavior, i.e., of metacognitive functioning, is indicated by higher scores, therefore, the presence of the ceiling effect is possibly explained by the non-clinical nature of the study population. Indeed, a scale for assessing affective disorders, i.e., the Hospital Anxiety and Depression Scale (HADS) was reported to show a floor effect when validated in a normal elderly Swedish population [36]. This situation is similar to the one we encountered for the MMSS, because in the case of the HADS, the lower score denotes normal behavior, while for the MMSS it is the higher score [36]. However, the absence of the ceiling/floor effect in the MMSS global score and factor scores, as well as the absence of the floor effect for all the MMSS item scores, are further evidence of its applicability in student populations [36]. Additionally, findings which were similar to our own with respect to the ceiling/floor effect were confirmed for the brief Meta-Cognition Questionnaire, of which the MMSS is an adapted version, thus providing concurrent evidence for the presently studied instrument’s overall validity [10, 24]. The Cronbach’s alpha if item deleted (all above 0.72) and item-Factor correlations (all above 0.75) indicate that the all items scores of the MMSS had favorable ability to discriminate between low and high scorers [37].

### Factor analysis

Though it is desirable to perform both exploratory factor analysis (EFA) and CFA for establishing factorial validity, it is also an acceptable practice to present findings from CFA for constructs based on theoretical considerations [10, 38]. Therefore, we employed CFA along with measurement invariance analysis across gender groups to evaluate the validity of the 2-Factor model of the MMSS.

### Measures assessing adequacy, suitability and factorability of the MMSS scores

Factor analysis was employed to investigate the scale’s dimensionality because the MMSS scores satisfied the conditions of sample adequacy, sample suitability, and factorability. Evidence for this conclusion came from findings such as the diagonal elements of the correlation anti-image matrix, Bartlett’s test of Sphericity, determinant, Kaiser-Meyer-Olkin Test of sampling adequacy (KMO), communality and inter-item correlations, all of which were within normal limits [32].

### Confirmatory factor analysis (CFA)

CFA was employed to establish the dimensionality conditions, though the instrument was expected to produce a 2-Factor model based on theoretical considerations [10]. Both models, i.e., the model-A, a2-Factor model and model-B, a 2-Factor model with incorporation of modification indices (correlated error terms) performed very similarly with excellent to acceptable values for the fit indices [28]. However, model-B was favored because of the higher value of the PClose and lower value of χ^2^/df. Furthermore, the very good to excellent level of correlations between the MMSS item scores and its factors for the model-B favor its validity [39].

### Measurement invariance of model-B among gender groups

Gender specific differences in metacognitive abilities are common in adolescents [40]. Moreover, gender dependent relationships between metacognitive dysfunctions and affective conditions such as anxiety and depression are also found among adults [41]. Given this background, it was imperative to assess that the MMSS construct comparability is not confounded by gender. Therefore, measurement invariance of the MMSS across gender groups was evaluated in the study population. The validity of the model-B, a 2-Factor model with incorporation of error terms was further evidenced by the establishment of its measurement invariance, i.e., configural, metric, scalar and strict invariance among two gender groups. For metric and scalar invariance, conditions for both, i.e., non-significant differences were found following chi-square testing and ΔCFI<.01 were met [35]. Even though the chi-square test of difference was significant the finding that ΔCFI<.01 still supports the strict invariance condition [35]. This is because ΔCFI is a more robust measure than chi-square test of difference [35].

### Internal consistency and homogeneity

According to the “rule of thumb” of [42] George and Mallery (2003), the MMSS and its subscale internal consistency were good, as implied by the Cronbach’s alpha and Mcdonald’s omega [42]. Furthermore, according to the criteria of Nunnaly and Bernstein, the Cronbach’s alpha of the factors of the MMSS suggest that it may have a potentially viable application in experimental research as well [22]. The Cronbach’s alpha of the MMSS was higher than that reported for the related instrument in a German elderly population (0.61–0.67) [10]. The internal homogeneity of the MMSS was supported by the strong item-total correlations in this student population. Here again, the item-total correlations were higher for the MMSS than that of the brief meta-cognition questionnaire in the German population (*r* = 0.26–0.52) [10]. Inter-item correlations indicated a moderate to a strong relationship, thus reinforcing the internal homogeneity of the MMSS in the study population.

### Divergent construct validity

Daytime sleepiness is an important defining feature of insomnia [43]. Furthermore, metacognition is associated with mental activity in primary insomnia [39, 40]. Therefore, ESS, which is a measure of sleepiness, was employed to assess the divergent validity of the MMSS. No correlation between the MMSS scores and the self-reported measure of daytime sleepiness support the divergent construct validity of the scale in the study population. This is because even though sleepiness and sleep are associated with meta-cognition in some populations but these represent non-overlapping constructs [44, 45]. In summary, the present findings of an absence of ceiling/floor effect for the MMSS global and factor scores, sufficient item discrimination, factorial validity, measurement invariance across gender groups for the factor structure of the MMSS, good internal consistency, strong internal homogeneity, and sufficient divergent validity favored psychometric validation of the MMSS in university students.

Some of the limitations of the study were that assessments of test-retest reliability, convergent validity, and concurrent validity were not carried out. The sample had a biased gender ratio. Therefore, the generalizations are more likely to be applicable for male students, who outnumbered females in the present study. Even though simple random sampling was used, fewer females completed the study, thereby causing the gender representation to be unbalanced. Future efforts to investigate the psychometric properties of the MMSS should accordingly anticipate and plan for a higher drop-out rate among female students, which could occur at any time from the stage of enrollment to the completion of the study. The scale was designed to assess to two important dimensions of the metacognition, i.e., meta-memory and meta-concentration. Future work should build on the current findings to incorporate brief subscales for other dimensions of metacognition to get a comprehensive yet brief tool to assess this function in students.

## Conclusion

Despite these qualifications, the findings of the present study are generally supportive of the value and applicability of this instrument. The MMSS, which is the first measure of meta-memory and meta-concentration to be evaluated in a sample of university students, thus has relevance for use in student populations. This conclusion is supported by psychometric measures of its ceiling/floor effect, internal consistency, internal homogeneity, divergent validity, factorial validity and measurement invariance of the validated factor structure across gender groups.

## Additional file

Additional file 1: Appendix I contains the Mizan meta-memory and meta-concentration scale for students (MMSS) and its scoring guideline. (DOCX 14 kb)

## Abbreviations

CFA: Confirmatory factor analysis
CFI: Comparative Fit Index
ESS: Epworth sleepiness scale
GFI: Goodness of fit index
HADS: Hospital Anxiety and Depression Scale
KMO: Kaiser-Meyer-Olkin Test of Sampling Adequacy
MCQ: Metacognition Questionnaire
MIA: Metamemory in Adulthood
MMSS: Mizan meta-memory and meta-concentration scale for students
RMSEA: Root mean square error of approximation
SRMR: Standardized root mean square residual
