# Supplementary material for: The Mizan meta-memory and meta-concentration scale for students (MMSS): a test of its psychometric validity in a sample of university students
Source: BMC Psychol. 2018 Dec 18;6:59. doi: 10.1186/s40359-018-0275-7 (PMC6299649; doi:10.1186/s40359-018-0275-7)
Supplement: Supplementary file 1 — Appendix I contains the Mizan meta-memory and meta-concentration scale for students (MMSS) and its scoring guideline. (DOCX 14 kb) [file 40359_2018_275_MOESM1_ESM.docx]

**Mizan Metamemory and meta-concentration scale for students (MMSS)**

**Brief Meta-memory Scale**

1. I have no trouble keeping track of my tasks like completing the assignments and preparation for the exams

Poor Fair Average Good Very Good

1 2 3 4 5

1. I am good at remembering conversations I had with my teachers/instructors and friends

Poor Fair Average Good Very Good

1 2 3 4 5

1. I have no trouble remembering where I have put my handouts and textbooks

Poor Fair Average Good Very Good

1 2 3 4 5

1. I am good at remembering the content of lectures and reproduce it well during exams and assessments

Poor Fair Average Good Very Good

1 2 3 4 5

1. I am usually able to remember exactly where I read or heard a specific concept related to a topic

Poor Fair Average Good Very Good

1 2 3 4 5

**Brief Meta-concentration Scale**

1. I am good at concentrating during lectures, workshops and practical attachments

Poor Fair Average Good Very Good

1 2 3 4 5

1. I am able to understand all the concepts taught from the start to the end during the class and practical attachments

Poor Fair Average Good Very Good

1 2 3 4 5

1. I have no trouble in keeping concentration during conversations with my professors and friends

Poor Fair Average Good Very Good

1 2 3 4 5

1. I am good at concentrating when reading subject textbook and listening to long lectures

Poor Fair Average Good Very Good

1 2 3 4 5
